# Visual Detection of West Nile Virus Using Reverse Transcription Loop-Mediated Isothermal Amplification Combined with a Vertical Flow Visualization Strip

**DOI:** 10.3389/fmicb.2016.00554

**Published:** 2016-04-20

**Authors:** Zengguo Cao, Hualei Wang, Lina Wang, Ling Li, Hongli Jin, Changping Xu, Na Feng, Jianzhong Wang, Qian Li, Yongkun Zhao, Tiecheng Wang, Yuwei Gao, Yiyu Lu, Songtao Yang, Xianzhu Xia

**Affiliations:** ^1^Key Laboratory of Jilin Province for Zoonosis Prevention and Control, Institute of Military Veterinary, Academy of Military Medical SciencesChangchun, China; ^2^Jiangsu Co-innovation Center for Prevention and Control of Important Animal Infectious Disease and ZoonosesYangzhou, China; ^3^Animal Science and Technology College, Jilin Agricultural UniversityChangchun, China; ^4^College of Veterinary Medicine, Jilin UniversityChangchun, China; ^5^Changchun SR Biological Technology Co., Ltd., ChangchunChina; ^6^Key Laboratory of Emergency Detection for Public Health of Zhejiang Province, Zhejiang Provincial Center for Disease Control and PreventionHangzhou, China

**Keywords:** West Nile virus, visual detection, reverse transcription loop-mediated isothermal amplification, visualization strip, RT-LAMP-VF

## Abstract

West Nile virus (WNV) causes a severe zoonosis, which can lead to a large number of casualties and considerable economic losses. A rapid and accurate identification method for WNV for use in field laboratories is urgently needed. Here, a method utilizing reverse transcription loop-mediated isothermal amplification combined with a vertical flow visualization strip (RT-LAMP-VF) was developed to detect the envelope (E) gene of WNV. The RT-LAMP-VF assay could detect 10^2^ copies/μl of an WNV RNA standard using a 40 min amplification reaction followed by a 2 min incubation of the amplification product on the visualization strip, and no cross-reaction with other closely related members of the *Flavivirus* genus was observed. The assay was further evaluated using cells and mouse brain tissues infected with a recombinant rabies virus expressing the E protein of WNV. The assay produced sensitivities of 10^1.5^ TCID_50_/ml and 10^1.33^ TCID_50_/ml for detection of the recombinant virus in the cells and brain tissues, respectively. Overall, the RT-LAMP-VF assay developed in this study is rapid, simple and effective, and it is therefore suitable for clinical application in the field.

## Introduction

West Nile virus (WNV) infection leads to an acute febrile zoonosis, which can cause disease in birds, humans and horses^1^ (Gubler, 2001). The manifestations of WNV infection include West Nile fever and West Nile encephalitis, which together comprise symptoms ranging from a benign or often symptomless infection to fever and neuroinvasive disease (Gubler, 2001; Sejvar et al., 2003; Hayes and O’Leary, 2004; Hayes and Gubler, 2006). WNV infection gained worldwide attention in 1999, when it was first detected in America during an outbreak in New York City (Nash et al., 2001; Hayes and Gubler, 2006). In America, as of January 12, 2016, a total of 48 states have reported WNV infection in humans, birds, or mosquitoes^2^.

WNV is an arthropod-borne, neurotropic, enveloped *Flavivirus* with a single-stranded, positive-sense RNA genome and is a member of the Japanese encephalitis virus (JEV) serogroup, which includes JEV, Murray Valley encephalitis virus (MVEV) and St. Louis encephalitis virus (Mukhopadhyay et al., 2003; Solomon et al., 2003; Lim et al., 2011). The RNA genome of WNV is approximately 11,000 nucleotides in length and encodes three structural [capsid (C), premembrane (prM) or membrane (M), and envelope (E)] proteins and seven non-structural (NS1, NS2a, NS2b, NS3, NS4a, NS4b, and NS5) proteins (Deubel et al., 2001; Brinton, 2002; Parida et al., 2004). Phylogenetic analysis of the WNV E protein sequence has indicated that WNV can be separated into two main lineages (lineages 1 and 2) as well as several additional minor lineages (lineage 3, lineage 4, lineage 5 and the putative lineage 6; Lanciotti et al., 2002; Vazquez et al., 2010; Del Amo et al., 2013; Faggioni et al., 2014).

There are many laboratory methods for diagnosing WNV, including virus isolation, immunohistochemistry, immunofluorescence, plaque reduction neutralization test (PRNT), enzyme-linked immunosorbent assay (ELISA), reverse transcription PCR (RT-PCR), real-time RT-PCR, and nested RT-PCR, among others (Dauphin and Zientara, 2007; De Filette et al., 2012). Some of these methods have been approved by the American Centers for Disease Control and Prevention (American CDC). However, the ELISA has cross-reactivity with other flaviviruses when used for serodiagnosis. Additionally, the above nucleic acid amplification methods are disadvantageous because they require expensive equipment and are time-consuming to perform. Furthermore, both virus isolation and PRNT are tedious and technically complex and require over a week to perform. Thus, there is an urgent need to develop a rapid and simple diagnostic method for WNV to improve detection efficiency.

Loop-mediated isothermal amplification (LAMP) is a novel technique that can amplify DNA with high efficiency, specificity and rapidity under isothermal conditions (Notomi et al., 2000). Because this amplification technique requires isothermal conditions, LAMP can be performed using only a simple water bath. Moreover, LAMP can detect RNA templates by using reverse transcriptase together with DNA polymerase for amplification (Whiting and Champoux, 1998; Notomi et al., 2000; Ge et al., 2013). Several RNA viruses have been successfully detected using RT-LAMP assays (Kurosaki et al., 2007; Ge et al., 2013). The gold immunochromatographic assay is another technique that has been widely applied for the detection of various viruses (Mikawa et al., 2009; Wang et al., 2010). Amplicon detection using a vertical flow (VF) visualization strip has been previously applied to accelerate and simplify the process of interpreting LAMP assay results (Cui et al., 2012). In the current study, we developed a RT-LAMP assay coupled with a VF visualization strip for rapid, simple, and accurate visual detection of WNV. Because of these characteristics, this RT-LAMP-VF assay is useful for field laboratory diagnosis of WNV infection.

## Materials and Methods

### Viruses and Extraction of Viral RNA

The recombinant rabies virus SRV9 strain was modified to express the E protein (EP) of WNV (GenBank Accession: DQ211652; rRABV-WNVE) and stored in our laboratory. The recombinant virus was propagated in BSR cells, a clone of the BHK-21 cell line (baby hamster kidney cells), which were grown in Dulbecco’s modified Eagle’s medium (DMEM, Gibco, Grand Island, NY, USA) supplemented with 5% fetal bovine serum (FBS, Gibco, Grand Island, NY, USA) at 37°C in an incubator. rRABV was titrated in Neuroblastoma (NA) cell as described previously (Wang et al., 2011). Viral RNA was extracted using a commercial RNA extraction kit (RNeasy Mini Kit, Qiagen, Hilden, Germany). All operations were performed according to the manufacturer’s instructions.

### Preparation of WNV RNA Standards

Artificial viral RNA encoding a partial WNV E gene sequence (GenBank Accession: DQ211652) was synthesized; this construct was modified via PCR to include the T7 promoter sequence at its 5′ terminus. The PCR products were transcribed *in vitro* using T7 RNA polymerase (TaKaRa Biotechnology Co., Ltd., Dalian, China) according to the manufacturer’s instructions. The RNA transcripts were then purified and quantified, and 10-fold serial dilutions of the RNA ranging from 10^7^ to 10^0^ copies per μl were prepared.

### Primer Design

**FIGURE 1:**
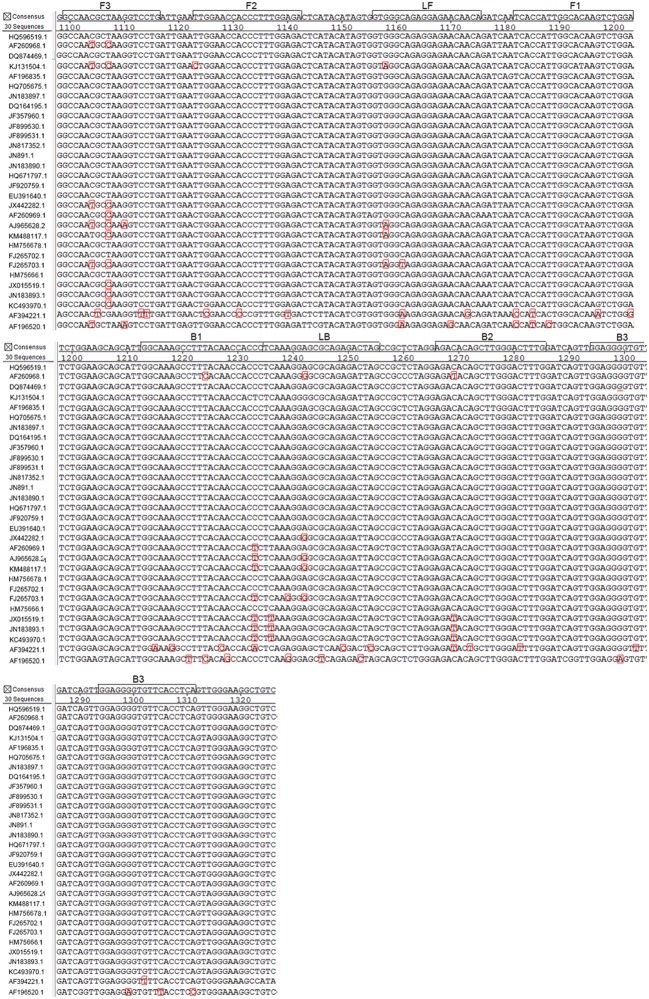
**Alignment of West Nile virus (WNV) E gene sequences and positions of the primers used for RT-LAMP.** The E gene of WNV was retrieved from GenBank and analyzed using MegAlign software. Primers were designed based on a conserved region (nucleotides 2065–2277 of the genome). The GenBank accession numbers of the aligned strains (from top to bottom) are HQ596519.1, AF260968.1, AF196520, KJ131504.1, AF196835.1, HQ705675.1, JN183897.1, DQ164195.1, JF357960.1, JF899530.1, JF899531.1, JN817352.1, JN891.1, JN183890.1,HQ671797.1, JF920759.1, EU391640.1, JX442282.1, AF260969.1, AJ 965628.1, KM488117.1, HM756678.1, FJ265702.1, FJ265703.1, HM756661.1, JX015519.1, JN183893.1, KC493970.1, AF394221.1, and AF196520.1.

**FIGURE 2:**
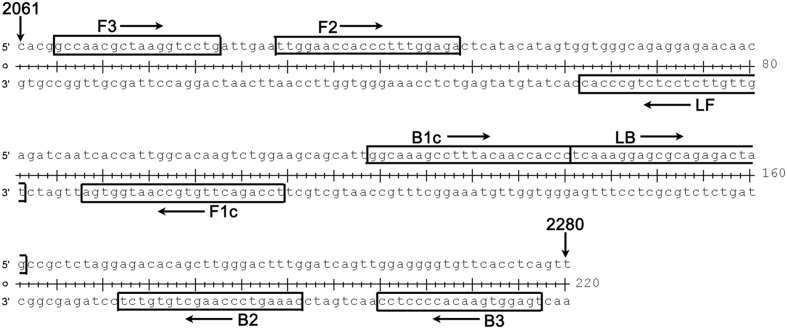
**Details (location and sequence) for each primer used in our RT-LAMP-VF assay.** The target region spanned nucleotides 2065–2277 of the complete genome of WNV strain NY99 (GenBank Accession: DQ211652); this region is located in the E gene of WNV.

The complete genome sequences of 30 strains of WNV isolated over various years from different regions and disparate species were analyzed, and the E gene of WNV was selected as the target region for the RT-LAMP-VF assay (the alignment of WNV E gene sequences was shown in **Figure 1**). Six primers, targeting eight distinct regions of the gene, were designed for the assay using the PrimerExplorer V4 program^3^. To detect reaction products by VF, the LF and LB primers were 5′-labeled with biotin and FITC. The details are shown in **Figure 2** and **Table 1**. All primers were synthesized by Sangon Biotech Biotechnology Co., Ltd., (Shanghai, China).

**Table 1:** Primer sequences used for the RT-LAMP-VF assay.

Primer name	Sequences (5′ to 3′)	Genome position
LAMP-F3	GCCAACGCTAAGGTCCTG	2065–2082
LAMP-B3	TGAGGTGAACACCCCTCC	2260–2277
LAMP-FIP (F1C + F2)	TCCAGACTTGTGCCAATGGTGA TTGGAACCACCCTTTGGAGA	F1C:2148–2169 F2:2089–2108
LAMP-BIP (B1C + B2)	GGCAAAGCCTTTACAACCACCC CAAAGTCCCAAGCTGTGTCT	B1C:2179–2200 B2:2232–2251
LAMP-LF^a^	Biotin-TGTTGTTCTCCTCTGCCCAC	2122–2141
LAMP-LB^b^	FITC-TCAAAGGAGCGCAGAGACTAG	2201–2221

*^a^The 5′ ends of the LAMP-LF was labeled with biotin. ^b^The 5′ ends of the LAMP-LB was labeled with FITC.*

### RT-LAMP-VF Reaction and Product Detection

The RT-LAMP-VF assay was performed using a 25-μl total reaction volume containing a mixture of 0.4 μM of the inner primers FIP and BIP, 0.2 μM of the loop primers LF and LB, 0.1 μM of the outer primers F3 and B3, 1.4 mM of each deoxynucleoside triphosphate (dNTP), 8 mM MgSO_4_, 0.2 M betaine, 5 U of avian myeloblastosis virus reverse transcriptase (Bioer Technology Co., Ltd., Hangzhou, China), 8 U of Bst DNA polymerase large fragment (New England BioLabs), and 5 μl of target RNA. The mixture was incubated at varying temperatures (58, 60, 62, 64, or 66°C) for 60 min, followed by heating at 80°C for 2 min to terminate the reaction. The mixture was incubated for different lengths of time (30, 40, 50 or 60 min) at the optimal temperature. In parallel with the optimization of the reaction time, two different concentrations were used: one group of the six primers was used at the concentration described above, and the other group was used at half this concentration. The RT-LAMP products were detected using a VF visualization strip (Ustar Biotech Co., Ltd., Hangzhou, China) as previously described (Cui et al., 2012).

### Specificity and Sensitivity of the RT-LAMP-VF Assay

RNAs of rRABV-WNVE (10^5^ TCID_50_/ml), JEV (10^5^ PFU/ml), dengue virus (DENV, 10^5^ PFU/ml), and classical swine fever virus (CSFV, 10^5^ TCID_50_/ml) were extracted using a commercial RNA extraction kit (RNeasy Mini Kit, Qiagen, Hilden, Germany). All operations were performed according to the manufacturer’s instructions, and the level of extracted RNAs was determined as 80–120 ng/μl. To evaluate the specificity of the RT-LAMP-VF assay, 5 μl of RNAs extracted from above viruses and synthesized RNA transcripts (10^7^ copies/μl) were added into the optimal reaction system.

The synthesized WNV RNA transcripts (1092 ng/μl) were purified, quantified, and prepared as standard samples with a concentration of 10^9^ copies/μl. Then, 10-fold serial dilutions of the standard samples (ranging from 10^7^ to 10^0^ copies/μl) were used to assess the detection limits of the RT-LAMP-VF assay.

### Evaluation of the RT-LAMP-VF Assay Using Live Virus RNA

Viral RNA extracted from rRABV-WNVE was used to further evaluate the performance ability of the RT-LAMP-VF assay. rRABV-WNVE at a titer of 10^6.5^ TCID_50_/ml was 10-fold serial diluted in DMEM. RNA was extracted from the dilutions, and the RNA samples were detected using the RT-LAMP-VF assay.

### Evaluation of the RT-LAMP-VF Assay Using Clinical Specimens

The feasibility of using the RT-LAMP-VF assay to detect WNV in clinical specimens was evaluated using 5-day-old and 3-week-old ICR mice. The mice were randomly divided into seven groups and intracerebrally infected with 10-fold dilutions of rRABV-WNVE ranging from 10^6^ to 10^1^ TCID_50_ per mouse. DMEM was used as a placebo. At 7 days post-infection, the mice were submitted to humane euthanasia by cervical dislocation under ketamine-xylazine anesthesia at a dose of 0.1 mL/10 g body weight, after which their brains were collected. The brain samples were then homogenized in phosphate-buffered saline (PBS, 10 mM, pH 7.2–7.4). The homogenates were centrifuged to remove debris, and the supernatants were collected for RNA extraction and virus titration.

Total RNA was extracted from the brain homogenate using an RNAeasy Mini Kit (Qiagen, Hilden, Germany). The extracted RNA was then assessed using the RT-LAMP-VF assay and real-time PCR as described previously (Yang et al., 2012). After viral titration, the brain homogenate with a high titer of 7.03 Log (TCID_50_/g tissue) was selected to evaluate the sensitivity of the assay. To accomplish this, RNA was extracted from a 10-fold serial dilution of the brain homogenate and measured using the RT-LAMP-VF assay; brain homogenate from healthy mice was used as a control.

### Ethics Statement

All animal studies performed in this work were approved by the Animal Care and Use Committee of the Chinese People’s Liberation Army (No. SYXK2009-045). All volunteers involved in this study provided written informed consent for the use of blood samples. All efforts were made to minimize animal suffering.

## Results

### Product Detection Using the RT-LAMP-VF Assay

**FIGURE 3:**
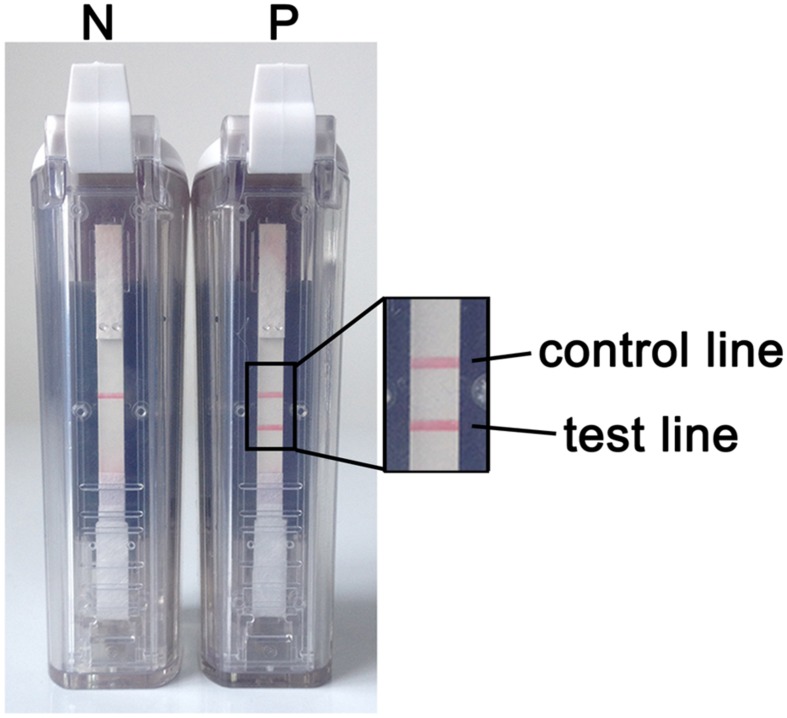
**RT-LAMP-VF assay results.** *N* denotes a negative result. In this case, only one red-purple band appears at the control line. *P* denotes a positive result. In this case, two red-purple bands appear: one at the test line and one at the control line.

When using the VF visualization strip, a clearly visible red-purple band at the control line was necessary for the test to be considered valid. The appearance of two red-purple bands at both the test and control lines was regarded as a positive result. If only the band at the control line appeared, then the result was considered negative (**Figure 3**).

### Optimization of RT-LAMP-VF Reaction Conditions

To determine the optimal conditions for the RT-LAMP-VF assay, synthesized RNA transcripts were used as a template to optimize both assay temperature and assay time. To determine the optimal temperature for the assay, 60-min reactions were performed at five different temperatures (58, 60, 62, 64, and 66°C). The results indicated that running the assay at 64°C produced the strongest amplification signal (**Table 2**); therefore, 64°C was considered the optimal temperature for the assay.

**Table 2:** Reaction temperature optimization for RT-LAMP^a^.

Temperature/°C	RNA dilution (Copies/μl)							
	10^7^	10^6^	10^5^	10^4^	10^3^	10^2^	10^1^	10^0^
58	+	+	+	+	-	-	-	-
60	+	+	+	+	-	-	-	-
62	+	+	+	+	+^b^	-	-	-
64	+	+	+	+	+	-	-	-
66	+	+	+	+	-	-	-	-

*^a^Three replications were performed for each trial. ^b^The result was weakly positive.*

To determine the optimal duration of time required for the RT-LAMP-VF assay, four different reaction times (30, 40, 50, or 60 min) were compared at 64°C. The best sensitivity and specificity were found when the reaction lasted for 40 min at 64°C (**Table 3**). Therefore, an amplification time of 40 min was selected as the optimal time for the assay.

**Table 3:** Reaction time optimization for RT-LAMP^a^.

Time/min	Primers group	RNA dilution (Copies/μl)							
		10^7^	10^6^	10^5^	10^4^	10^3^	10^2^	10^1^	10^0^
30	1^b^	+	+	+^d^	+^d^	-	-	-	-
	2^c^	+	+	+	+	-	-	-	-
40	1	+	+	+	+	+	+	-	-
	2	+	+	+	+	-	-	-	-
50	1	+	+	+	+	+	-	-	-
	2	+	+	+	+	-	-	-	-
60	1	+	+	+	+^d^	-	-	-	-
	2	+	+	+	+	+	-	-	-

*^a^Three replications were performed for each trial. ^b^The six primers was used as described as bellow: 0.4 μM each of inner primers FIP and BIP, 0.2 μM loop primers LF and LB, 0.1 μM outer primers F3 and B3. ^c^The six primers was used as described as bellow: 0.2 μM each of inner primers FIP and BIP, 0.1 μM loop primers LF and LB, 0.05 μM outer primers F3 and B3. ^d^The result was weakly positive.*

As shown in **Table 3**, primer concentration was also optimized when optimizing the reaction time. The following primer concentrations were considered optimal: 0.4 μM for the inner primers FIP and BIP, 0.2 μM for the loop primers LF and LB, and 0.1 μM for the outer primers F3 and B3.

### Specificity of the RT-LAMP-VF Assay

**FIGURE 4:**
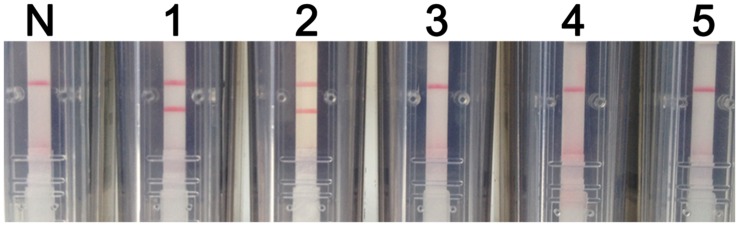
**RT-LAMP-VF assay specificity.** The specificity of the RT-LAMP-VF assay was determined by analyzing synthesized RNA transcripts and RNA extracted from rRABV-WNVE, JEV, DENV, and CSFV. N: blank control; 1: synthesized RNA transcripts; 2: rRABV-WNVE RNA; 3: JEV RNA; 4: DENV RNA; 5: CSFV RNA.

The analytical specificity of the RT-LAMP-VF assay was determined using synthesized RNA transcripts and RNA extracted from control viruses, including JEV, DENV, and CSFV. As shown in **Figure 4**, all of the tested control samples appeared identical to the blank control and produced only one red–purple band at the location of the control line; therefore, all of these samples produced negative test results. A positive test line was observed when using synthetic WNV RNA transcripts and RNA extracted from rRABV-WNVE as templates. Thus, because the RT-LAMP-VF assay had no cross-reactivity with other viruses related to WNV, the assay was considered to have high specificity.

### Sensitivity of the RT-LAMP-VF Assay

**FIGURE 5:**
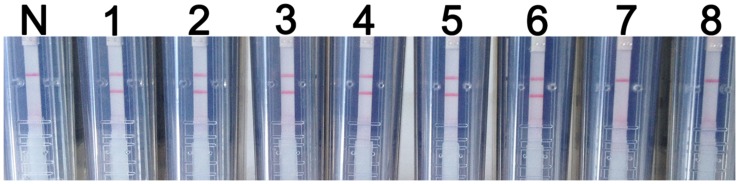
**RT-LAMP-VF assay sensitivity.** The sensitivity of the RT-LAMP-VF assay was analyzed by using tenfold serial dilutions of synthesized RNA transcripts. N: blank control; 1: 10^7^ copies/μl; 2: 10^6^ copies/μl; 3: 10^5^ copies/μl; 4: 10^4^ copies/μl; 5: 10^3^ copies/μl; 6: 10^2^ copies/μl; 7: 10^1^ copies/μl; 8: 10^0^ copies/μl.

Synthetic WNV RNA transcripts were 10-fold serially diluted to create samples ranging in concentration from 10^7^ to 10^0^ copies per μl. These samples were then used to assess the sensitivity of the RT-LAMP-VF assay. After a 40-min amplification reaction, negative results were produced at concentrations lower than 10^2^ copies/μl. Thus, the RT-LAMP-VF assay had a detection limit of 10^2^ copies/μl of synthetic RNA (**Figure 5**).

### Evaluation of the RT-LAMP-VF Assay Using Live Virus RNA

**FIGURE 6:**
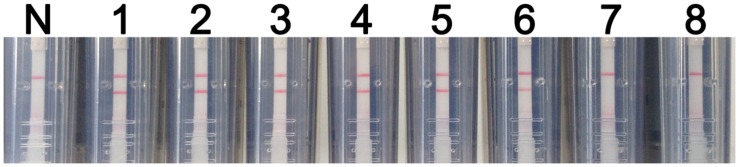
**Evaluation of the RT-LAMP-VF assay using live virus.** The sensitivity of the RT-LAMP-VF assay was analyzed by using 10-fold serial dilutions of recombinant virus (rRABV-WNVE). N: blank control; 1: 10^6.5^ TCID_50_/ml; 2: 10^5.5^ TCID_50_/ml; 3: 10^4.5^ TCID_50_/ml; 4: 10^3.5^ TCID_50_/ml; 5: 10^2.5^ TCID_50_/ml; 6: 10^1.5^ TCID_50_/ml; 7: 10^0.5^ TCID_50_/ml; 8: 10^-0.5^ TCID_50_/ml.

Total RNA was extracted from serial dilutions of rRABV-WNVE and then assessed using the RT-LAMP-VF assay. As shown in **Figure 6**, RNA extracted from a 10^5^-fold dilution of virus (10^1.5^ TCID_50_/ml) produced positive results when assayed; thus, the detection limit of the assay for rRABV-WNVE in cell culture is 10^1.5^ TCID_50_/ml. To confirm the absence of non-specific reactions, total RNA extracted from DMEM and NA cells was also evaluated; no amplification was found in either case.

### Evaluation of the RT-LAMP-VF Assay Using Clinical Specimens

Brain tissues collected from mice infected with rRABV-WNVE were used as clinical specimens to further evaluate the RT-LAMP-VF assay. As shown in **Table 4**, rRABV-WNVE mRNA in the range of 6.14–10.02 Log(copy number/g tissue) was extracted from the brains of rRABV-WNVE-infected suckling mice, corresponding to viral titers in the range of 4.31–7.35 Log(TCID_50_/g tissue); the RT-LAMP-VF results were positive across this entire range. Unlike the suckling mice, substantially less rRABV-WNVE mRNA was extracted from the brains of 3-week-old mice brain: from 2.16 to 5.56 Log(copy number/g tissue) mRNA was extracted, and two of the samples from the 10^2^ group were not detected. Thus, viral titers could not be determined for the 3-week-old mice; however, all of the RT-LAMP-VF results were positive, except for three samples in the 10^1^ and 10^2^ groups (**Table 5**).

**Table 4:** Assay detection results from suckling mouse brain tissue samples.

Samples^a^		mRNA [Log (copy number/g tissue)]	Virus titers [Log (TCID_50_/g tissue)]	RT-LAMP
Blank		Not detected	Not detected	-
Negative 1		Not detected	Not detected	-
Negative 2		Not detected	Not detected	-
10^1^	1	8.05	5.32	+
	2	7.21	6.67	+
	3	9.59	5.67	+
	4	6.14	6.66	+
10^2^	1	9.29	6.15	+
	2	10.02	7.03	+
	3	9.28	7.27	+
	4	7.30	6.18	+
10^3^	1	8.93	5.46	+
	2	8.46	6.24	+
	3	8.14	6.33	+
	4	7.65	5.68	+
10^4^	1	7.58	6.18	+
	2	9.45	5.09	+
	3	7.35	7.21	+
	4	6.51	6.16	+
10^5^	1	8.96	6.62	+
	2	8.79	4.31	+
	3	7.48	7.35	+
	4	7.17	7.34	+
10^6^	1	8.38	5.22	+
	2	8.28	6.65	+
	3	9.27	4.35	+
	4	8.82	6.23	+

*^a^10^1^, 10^2^, 10^3^, 10^4^, 10^5^, 10^6^ are the virus dose which was infected in mice (TCID_50_/mouse); 1, 2, 3 are different numbers of mouse in each group.*

**Table 5:** Assay detection results from 3-week-old mouse brain tissue samples.

Samples^a^		mRNA [Log (copy number/g tissue)]	Virus titers [Log (TCID_50_/g tissue)]	RT-LAMP
Blank		Not detected	Not detected	-
Negative 1		Not detected	Not detected	-
Negative 2		Not detected	Not detected	-
10^1^	1	2.36	Not detected	+
	2	4.11	Not detected	+
	3	2.16	Not detected	-
10^2^	1	Not detected	Not detected	-
	2	Not detected	Not detected	-
	3	2.49	Not detected	+
10^3^	1	4.73	Not detected	+
	2	3.02	Not detected	+
	3	5.11	Not detected	+
10^4^	1	3.23	Not detected	+
	2	5.76	Not detected	+
	3	3.52	Not detected	+
10^5^	1	3.53	Not detected	+
	2	4.00	Not detected	+
	3	4.13	Not detected	+
10^6^	1	4.68	Not detected	+
	2	4.97	Not detected	+
	3	5.56	Not detected	+

*^a^10^1^, 10^2^, 10^3^, 10^4^, 10^5^, 10^6^ are the virus dose which was infected in mice (TCID_50_/mouse); 1, 2, 3, 4 are different numbers of mouse in each group.*

The sensitivity of the RT-LAMP-VF assay was further evaluated using brain homogenates collected from rRABV-WNVE-infected mice; brain homogenates from uninfected mice were used as a control. After diluting the rRABV-WNVE-infected brain homogenates by 10^5^-fold, the RT-LAMP-VF assay still produced positive results. Therefore, the sensitivity of the assay for rRABV-WNV-infected mouse brain tissue is 10^1.33^ TCID_50_/ml.

## Discussion

With the unexpected appearance of WNV in many countries, it is becoming increasingly important to develop adequate surveillance methods for WNV. Such methods are needed not only in countries where WNV infections are epidemic but also in countries threatened by WNV infection and even in countries where WNV infection has not yet spread. In addition, the development of a rapid and reliable method for diagnosing WNV is critically important for the prevention of infection, the implementation of appropriate countermeasures, and the optimization of healthcare resources. In the current study, we described the development of a RT-LAMP-VF assay that provides a simple and rapid method to detect WNV infection.

To accomplish the above, we analyzed the complete genome sequences of 30 strains of WNV isolated over various years from different regions and disparate species, including representative strains from WNV lineages 1, 2, and 3. Bases on this analysis, we selected two target regions for RT-LAMP-VF, a region located in the E gene and a region located in the 5′-UTR and spanning part of the C gene. However, when using the RT-LAMP-VF assay to detect the latter region, the assay produced low sensitivity (data not shown). Several previously reported molecular biology methods have used the E gene as a target sequence with excellent results, consistent with our previous study (Parida et al., 2004; Zink et al., 2013; Kumar et al., 2014). Therefore, we chose a relatively conserved region of the genome spanning nucleotide positions 2065–2277 as a target sequence.

We designed a set of six primers (including two loop primers) targeting eight regions located in the conserved region of the WNV E gene to ensure high specificity for nucleic acid amplification. The use of loop primers accelerated the reaction time, enabling the reaction to be completed in less than half the time required for our original LAMP assay (Nagamine et al., 2002). Indeed, amplification could be completed within 40 min, which is faster than other molecular biology methods.

Compared to other molecular biology detection technologies, the RT-LAMP-VF assay is economical, technically simple, and rapid. For this assay, reverse transcription and cDNA amplification are performed in a single step, and no additional procedures are needed. Moreover, some conventional molecular biology methods, such as RT-PCR, nested RT-PCR and real-time PCR, have inherent flaws; in particular, specialized PCR instruments are needed, and temperature must be precisely controlled for a lengthy period of time. This inhibits the application of these methods in rural and remote areas (Deng et al., 2015). As the reaction conditions for RT-LAMP-VF are isothermal, only a water bath is needed to complete the assay, offering a measure of practicality for field laboratories in economically impoverished areas.

Parida et al. (2004) developed a RT-LAMP method for the rapid detection of WNV. In this method, the results were analyzed using agarose gel electrophoresis or real-time turbidity analysis (Parida et al., 2004). Generally, the use of gel electrophoresis to detect amplification products increases the risk of product contamination and degradation, while real-time turbidity analysis suffers from background interference (Iwamoto et al., 2003; Ge et al., 2013). In the current study, we modified the RT-LAMP technique by using two loop primers labeled with FITC and biotin. This allowed labeled amplification products to be analyzed using a VF visualization strip housed inside of an enclosed, plastic, leak-proof device, without the need for an electrophoresis apparatus or a turbidimeter. Within the strip, the FITC-labeled amplification products can bind to the anti-FITC antibody located on the test line, and the biotin-labeled amplification products are captured by colloidal gold particles conjugated to the anti-biotin antibodies (Chow et al., 2008; Cui et al., 2012). Using this strategy, we reduced not only the reaction time but also the chances of product contamination. Moreover, the results of the assay can be directly visualized within 2 min, and the plastic device was designed to prevent leakage of the amplification products.

As encephalitis is the main clinical symptom of WNV infection, we used brain samples from *Charadrius alexandrinus* and *Recurvirostra avosetta* to evaluate the specificity of the RT-LAMP-VF assay. All of the samples were negative (data not shown). Clinical specimens infected with WNV (e.g., blood and brain tissue) are difficult to obtain in non-endemic countries such as China; however, we hypothesized that using a recombinant virus expressing the E gene of WNV would produce similar assay results. Therefore, we assessed brain tissues collected from rRABV-WNVE-infected mice as clinical specimens in this study. Our results indicated that the assay could successfully detect clinical samples infected with the recombinant virus. Additionally, the assay thresholds for detecting cell culture samples infected with the recombinant virus (sensitivity: 10^1.5^ TCID_50_/ml) and mouse brain tissues infected with the recombinant virus (sensitivity: 10^1.33^ TCID_50_/ml) were nearly identical, which indicated that the presence of tissue-specific RNA did not influence the performance of the RT-LAMP-VF assay. Furthermore, three of the samples collected from the 3-week-old ICR mice had very little rRABV-WNVE mRNA, and the RT-LAMP-VF results were negative for these samples. This result indicates that the assay’s results may be ambiguous in cases of very small viral loads. Under such conditions, sample detection should be performed in duplicate or combined with other methods. To simulate brief viremia, blood samples from healthy volunteers were mixed with rRABV-WNVE and evaluated using the RT-LAMP-VF assay. In this case, similar results were produced as those generated by the rRABV-WNVE-infected mouse brain tissues (data not shown). The broad capacity to accurately identify the presence of WNVE in clinical specimens validated the RT-LAMP-VF assay for the detection of WNV infection.

In summary, we developed a method to diagnose WNV using isothermal amplification combined with a VF visualization strip. This method was shown to have high specificity and high sensitivity for the detection of WNV. To simulate clinical samples, brain tissue infected with a recombinant virus expressing the E gene of WNV was evaluated. The results showed that the assay could accurately detect the virus with no interference from tissue-specific RNA. Furthermore, in the clinic, viral RNA loads reach higher levels than the detection limit of our assay (Ramos et al., 2012; Caraballo et al., 2015). Collectively, the above results demonstrate that our RT-LAMP-VF assay offers rapid, simple and effective diagnostic identification of WNV infection.

## Author Contributions

HW, SY, and XX designed the experiments. ZC, LW, LL, HJ, CX, FY, JW, QL, YZ, and TW performed the experiment. ZC, HW, LL, YG, YL, SY, and XX analyzed the data. ZC and HW wrote the manuscript. All authors reviewed the manuscript.

## Conflict of Interest Statement

The authors declare that the research was conducted in the absence of any commercial or financial relationships that could be construed as a potential conflict of interest.

## Footnotes

1 http://www.oie.int/en/for-the-media/animal-diseases/animal-disease-information-summaries/

2 http://www.cdc.gov/westnile/index.html

3 http://primerexplorer.jp/elamp4.0.0/index.html
